# Visual Saliency Prediction and Evaluation across Different Perceptual Tasks

**DOI:** 10.1371/journal.pone.0138053

**Published:** 2015-09-14

**Authors:** Shafin Rahman, Neil Bruce

**Affiliations:** Department of Computer Science, University of Manitoba, Winnipeg, Manitoba, Canada; UMR8194, FRANCE

## Abstract

Saliency maps produced by different algorithms are often evaluated by comparing output to fixated image locations appearing in human eye tracking data. There are challenges in evaluation based on fixation data due to bias in the data. Properties of eye movement patterns that are independent of image content may limit the validity of evaluation results, including spatial bias in fixation data. To address this problem, we present modeling and evaluation results for data derived from different perceptual tasks related to the concept of saliency. We also present a novel approach to benchmarking to deal with some of the challenges posed by spatial bias. The results presented establish the value of alternatives to fixation data to drive improvement and development of models. We also demonstrate an approach to approximate the output of alternative perceptual tasks based on computational saliency and/or eye gaze data. As a whole, this work presents novel benchmarking results and methods, establishes a new performance baseline for perceptual tasks that provide an alternative window into visual saliency, and demonstrates the capacity for saliency to serve in approximating human behaviour for one visual task given data from another.

## 1 Introduction

For many saliency algorithms, the goal is to approximate fixation locations in eye tracking data derived from many human observers. An assumption attached to this analysis, is that fixation points capture loci of attention, which are assumed to correspond to salient targets in the scene in the absence of a specific task. However, there are known sources of bias present in eye tracking data (e.g. central or peripheral spatial bias [1]). There is also undoubtedly variation in the data that derives from the nature of eye movements themselves, including physical and neural constraints on oculomotor control, error due to imprecise targeting, and inherent spatial dependencies across fixations. Thus, an observed fixation point does not guarantee that the corresponding location corresponds to a target of interest within the scene. As an alternative window into content deemed to be perceptually salient by humans, Koehler *et al*. [2] proposed an alternative task called explicit perceptual judgment. In this task, locations selected as salient are based on a manual selection of the most salient location in a scene, and there is no measurement of eye movements. In collecting data of this type, a larger number of human participants is required to derive a suitably sized pool of data, and due to individual variation a variety of locations within a scene will typically be selected as most salient across observers. Explicit judgment does not carry the same spatial bias inherent in fixation data, however there remains spatial bias in image content. In many instances, explicitly chosen points correspond to objects in a scene, which implies a process more closely related to early proto-object selection. Therefore, algorithms that predict explicit judgment locations may be more appropriate as an early stage of processing in object segmentation or recognition, and related tasks.

Saliency maps produced by predictive algorithms might therefore carry the goal of approximating locations selected via explicit judgment rather than fixations in eye tracking data for the reasons mentioned. We also show in this paper, that explicit judgment locations may carry similar information to fixated locations but in the absence of some of the noise sources inherent in eye movement data. Although a variety of benchmarking efforts have been undertaken by the research community to evaluate saliency algorithms in predicting fixation data [3, 4], only very limited benchmarking has been carried out for explicit judgment approximation [2] on 3 existing algorithms. One contribution of this paper is to present benchmarking results for a wider range of algorithms to assess their efficacy in predicting explicit judgment locations. In line with the results of Koehler *et al*. [2], we observe that existing saliency algorithms agree to a much greater extent with locations selected through explicit judgment than through free viewing. In the context of this analysis, we also relate the spatial locations selected across these different task definitions to estimate their similarity.

The similarity between fixation data and explicit judgment data that we demonstrate, suggests overlap in factors that drive these two processes. To gain further insight into this relationship, we measure the extent to which locations corresponding to explicit judgments may be predicted by the fixation data for different tasks. This investigation establishes that explicit judgment coordinates may be approximated to some extent by the different classes of eye tracking data. We also improve upon the state-of-the-art in performance for predicting explicit judgments, using the ensemble output across a range of saliency algorithms. Given that eye tracking data is a relatively standard measurement and will continue to be in many application areas, we also consider whether explicit judgments associated with an image might be approximated from fixations, or saliency. That is, if one has only eye tracking data available, can this be converted into an approximation of behavior for the explicit judgment task for the same image? In this manner, data collected to probe one cognitive process, may be re-purposed to allow qualitative and quantitative analysis consistent with an alternative cognitive process. In the current work, we demonstrate the relative value of fixation data, and saliency output in making this prediction. This demonstrates the relatedness of data derived from different visual processes, and importantly shows that i. Eye tracking data may be used to simulate explicit judgment data when the latter is unavailable. ii. This simulation is of sufficient quality to approximate relative performance of algorithms in predicting explicit judgment, even if only fixation data is available.

In producing the benchmark results, a number of nuances related to the data are considered. Among these, we propose a method for performance evaluation that corrects for data bias in a manner that is distinct from prior work. This is shown to produce greater consistency in evaluation results, and also controls for spatial bias in fixation data, and non-uniform importance weighting inherent in existing evaluation methods.

## 2 Benchmarking of explicit judgment

In viewing an image, an observer will fixate on locations in the image by their own volition, due to the nature of stimulus patterns or other external factors. Rather than considering fixated locations, an alternative is having participants in a user study select a specific location of interest. This presents an additional quantity of value for saliency algorithms to predict. This final selected location is called the explicit judgment of the observer corresponding to the most salient location. Saliency models typically attempt to predict the gaze points in fixation data rather than explicit judgments. The recent study of Koehler *et al*. [2] addresses this shortcoming, providing a rich dataset containing explicit judgments for 100 human observers. Apart from the explicit judgment task, Koehler et al. also considered three other tasks (free viewing, saliency viewing and a cued object search task). The free viewing task represents searching behavior without any particular goal, which is the scenario traditionally considered in most datasets in the literature. In the saliency search task, observers were asked to decide whether the right or left portion of the image is more salient than the other. Finally, in the object search task, observers were instructed to find some pre-defined objects. Except for the explicit judgment case, each of these tasks is examined through eye tracking experiments.

Relative to fixation data, very little evaluation has focused on the ability of saliency algorithms to approximate locations judged as salient in an explicit judgment task. Although Koehler *et al*. [2] presented results in this regard, only a very narrow range of algorithms were considered, and therefore there is value in considering a wider range of established algorithms. In Table 1 we have reported benchmark results for 12 algorithms including area under the curve (AUC) scores from ROC analysis for all four tasks: free viewing, saliency viewing, cued object search and explicit judgment tasks. Those algorithms are Torralba [5], HouCVPR [6], HouNIPS [7], Itti-CIO2 [8], ImageSignatureLab [9], ImageSignatureRGB [9], SDSR [10], AIM [11], GBVS [12], AWS [13], Yan [14] and SOC [15]. In AUC based evaluation, we have considered two different types of ROC analysis: Standard ROC [1, 3] and shuffled ROC [4, 16] that each apply a signal detection approach to evaluate the prediction performance of saliency maps, but that treat spatial bias differently. As may be seen from Table 1, there is some consistency in the performance of algorithms, but also significant variability dependent on the specific nature of ROC analysis. In results that follow, we show that both standard and shuffled ROC analysis may be affected by different types of data bias.

**Table 1:** Benchmarking performance for the Koehler *et al*. [2] dataset.

Task	Free Viewing		Object Search		Saliency Viewing		Explicit Judgment	
AUC	Standard	Shuffled	Standard	Shuffled	Standard	Shuffled	Standard	Shuffled
HouCVPR [6]	.726	.639	.709	.632	.735	.652	.735	.657
SOC [15]	**.786**	.632	.722	.630	.742	.643	.736	.643
HouNIPS [7]	.719	.626	.700	.614	.733	.644	.748	.674
ImageSignatureRGB [9]	.739	.639	.725	.634	.750	.652	.749	.657
Itti-CIO2 [8]	.753	.629	.743	.638	.754	.638	.758	.654
Torralba [5]	.769	.635	.749	.629	.766	.642	.763	.652
SDSR [10]	.747	.643	.727	.635	.756	.657	.768	.683
ImageSignatureLab [9]	.746	.642	.727	.633	.756	.659	.773	.688
AIM [11]	.753	.632	.733	.624	.756	.640	.774	.665
AWS [13]	.742	**.645**	.719	.632	.709	**.664**	.778	**.699**
GBVS [12]	.782	.633	**.772**	**.649**	**.776**	.635	.781	.655
Yan [14]	.769	.644	.753	.647	.775	.656	**.782**	.682
IOC score [17]	.847	.716	.875	.770	.847	.724	-	-

Another relevant observation based on the results appearing in Table 1 is that AUC scores for both standard and shuffled evaluation metrics are relatively close across different algorithms for all tasks except for the free viewing case. This may reflect a bias in the degree of bottom-up and top-down guidance reflected across different tasks. Free viewing data may be assumed to be most strongly driven by bottom-up processing and characterized by a relative lack of influence from contextual guidance or prior experience. Inter observer congruency (IOC) scores at the last row of Table 1 are also consistent with this observation which is considered as upper bound of performance [17]. The commonality in viewing patterns is lower for the free viewing case than object search or saliency viewing. For this reason, the potential for variability of algorithm performance is higher.

### 2.1 The impact of center bias

In a free viewing task, observers have a tendency to look at the middle of an image or scene, a tendency referred to as center-bias. Tatler [1] discussed in detail the cause and effect of center bias in human eye tracking data. He showed that in free viewing, center bias is present even if salient objects are not within the center of the scene. Reasons for center bias include motor biases in the saccadic system, the distribution of image features, prior bias in the viewing strategy of subjects, the specifics of the experimental setup, and other situational factors [1, 4, 16]. To account for spatial (centre) bias in fixation data, algorithm output is often re-weighted by a centrally located Gaussian profile to better predict fixated regions in the data. However, there are different degrees of systematic spatial bias in the output produced by different algorithms. Since different saliency algorithms exhibit different degrees of center bias, this poses a challenge for producing a fair comparison across algorithms [4]. To address this problem, Zhang *et al*. [16] proposed a metric for evaluation of saliency algorithms called shuffled AUC. In ROC analysis, this evaluation method chooses positive and negative samples in a manner that removes the effect of center bias, by selecting negative samples from a spatial distribution that matches fixations within the entire dataset. However, as a side-effect, this may result in uneven importance of pixel locations in evaluation. Given a much larger number of centrally located samples across the dataset, the importance of saliency output at the center is relatively diminished given that there are likely to be more negative samples within the center region. This diminishes the capacity for an above chance prediction in a signal detection theoretic sense as a function of spatial position. Moreover, a relative bias outside of the center allows for the true positive rate to grow more quickly than the false positive rate due to the spatial distribution of negative samples. This issue has not yet been addressed in existing work. In this paper, we propose a method to control for bias in both algorithm output and data such that all pixel locations are equally important, and algorithms have no spatial bias.

### 2.2 Approximation of explicit judgments

Gaze patterns in the absence of an explicit task (free viewing) and with a prior task (object search, saliency viewing) are both experimental paradigms that have been widely studied in the literature [3, 18]. In contrast, the more direct process of making a manual selection proposed by Koehler *et al*. [2] diverges from the traditional methods for examining the visual selection process through eye tracking. Koehler *et al*. argued that algorithmic determination of saliency bears a closer resemblance to locations selected through explicit judgment than fixation data collected from any of the other three tasks. In the current work, this suggestion is confirmed in Table 1, with further information on the performance landscape for different popular algorithms across the different tasks.

We believe that improving the prediction of explicit judgments is likely more prudent than improvements to performance for the traditional fixation tasks for several reasons: Explicit judgment captures the most salient locations within a scene, through a selection process that is less clouded by noise from spatial bias and fixation mechanisms, and with more relation to content relevant to the role of saliency as in applications in computer vision and multimedia. Although there is evidently value in focusing on explicit judgment prediction, this has not been on the radar of development in algorithms targeting visual saliency, and also there is a relative paucity of this type of data. Moreover, in the future the tradition of examining perception through eye tracking across many areas of study is likely to continue. In this paper, we therefore strive to present a means of simulating explicit judgment data via saliency and eye tracking data. This allows for broader capability in the qualitative analysis that explicit judgment provides, and also as a means of providing a larger corpus of simulated explicit judgment data to drive improvements in visual saliency prediction.

### 2.3 Comparison of tasks in terms of IOC

**Fig 1:**
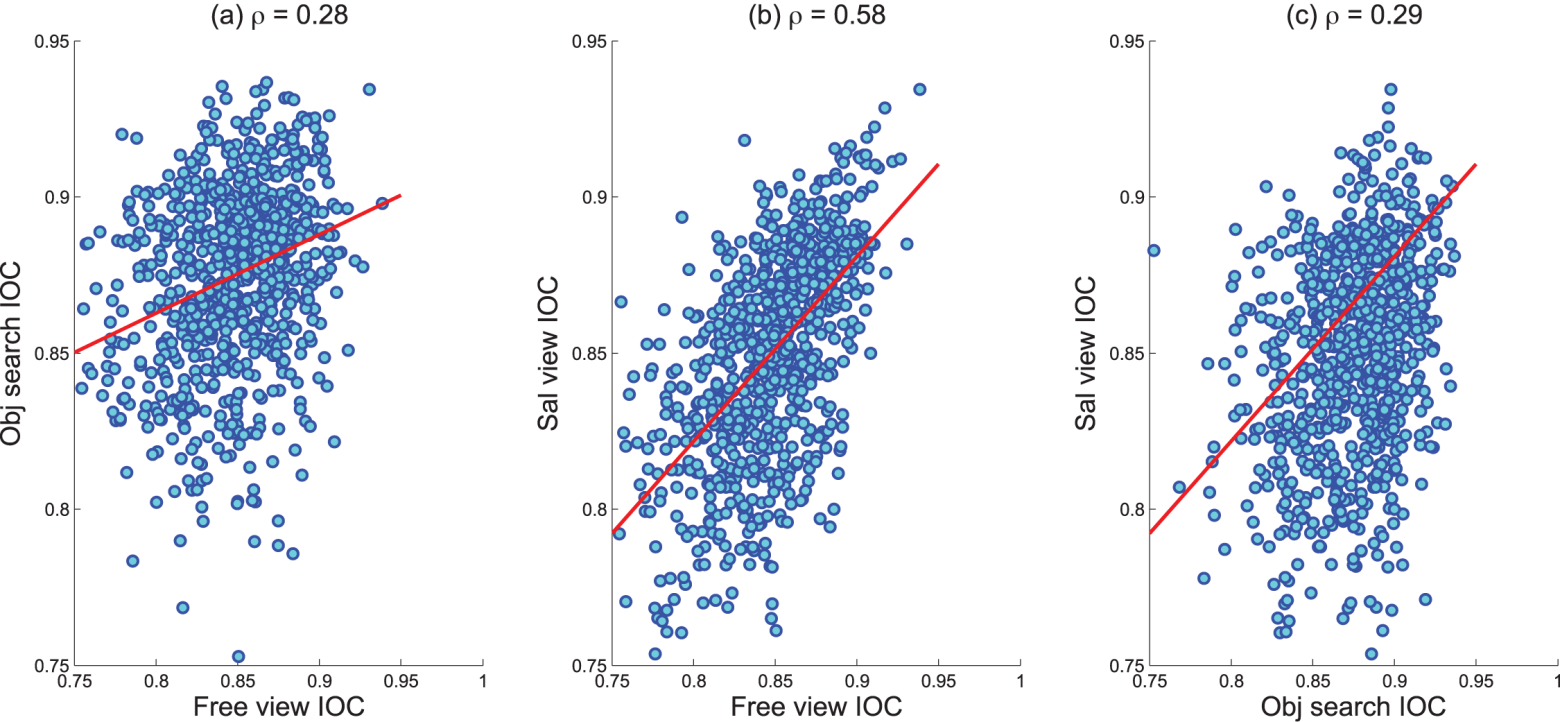
Scatter plot showing the correlation among the IOC of free view, obj. search and sal. view tasks.

The viewing pattern of observers varies from task to task. Being motivated by bottom up saliency, the free viewing task is meant to capture the task-independent bias in viewing the image. All fixation data also carries an implicit tendency to look at the center of the scene [1]. Such center bias is present even when stimuli are not placed at the center of the image. Unlike the free viewing task, when contextual guidance is imposed, fixated locations more closely follow the regions where target objects are present and center bias is somewhat diminished [16]. This scenario occurs to a significant extent for the object search task and also occurs to a lesser extent for the saliency viewing task. Because of the differences in viewing pattern, the agreement among the observers varies from one task to another. To observe this variation, we have calculated the correlation among the inter observer congruency (IOC) scores of different types of task presented in Koehler *et al*. [2] dataset. Traditionally, the IOC score is a single value calculated for an entire dataset [17] and this value is also presented in Table 1 for reference. To examine this quantity in greater detail, we have calculated IOC scores for individual images and found correlation for IOC scores among different tasks on a per-image basis. The correlation between free view vs. object search, free view vs. saliency view and object search vs. saliency view are 0.28, 0.58 and 0.29 respectively. The scatter plot of these measurements is presented in Fig 1. These results suggest that the agreement in viewing pattern across observers for free viewing correlates with agreement for saliency viewing to a greater extent than other task combinations. This also suggests overlap in the underlying factors driving gaze selection for these two conditions. In the object search task, the distinction between bottom-up and top-down factors in viewing evidently plays a greater role, lending some credence to the bottom-up or stimulus driven claim that is typically attached to free viewing fixation data. With that said, it is evident that there are significant differences between any of the fixation based measurements and the explicit judgment condition, and we shed further light on this point in the sections that follow.

### 2.4 Relation of explicit judgment to IOC in fixation tasks

**Fig 2:**
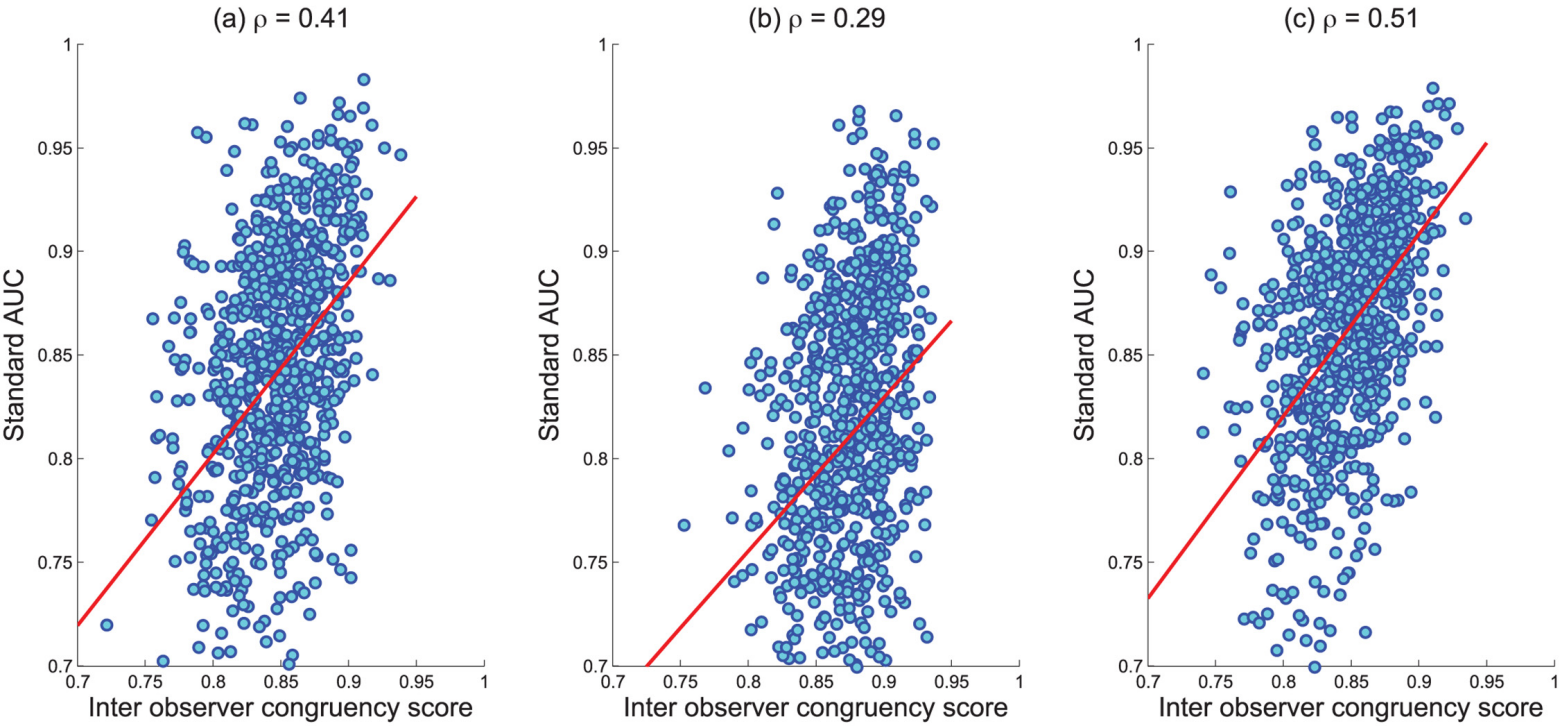
Scatter plot showing the correlation between Standard AUC of explicit judgment approximation vs. IOC scores of (a) free view (b) object search (c) saliency view fixation data.

If there is strong agreement in viewing pattern across observers for fixation data across different tasks, one might intuit that overlapping locations may be especially salient. One might also suspect that such locations may be among those selected in the explicit judgment task. Therefore, we hypothesize that a high IOC score for an image may be indicative of instances wherein a fixation map makes a good approximation of explicit judgment. To test this hypothesis, we have calculated correlation between the standard AUC performance of the fixation maps in predicting locations selected via explicit judgment, and the corresponding IOC score for a given image within-task. The scatter plot is shown in Fig 2. We find that the correlation for the object search task is lower than that of free viewing and saliency viewing tasks. In conjunction with the results reported in Table 2 of the main paper, this analysis hints at the relatedness of the different task conditions. Evidently, there is some top-down influence within each of these tasks, and influence from cognitive processes unrelated to saliency. However, that algorithms bear a stronger resemblance to explicit judgment data when the IOC score is high strongly suggests that these processes converge, and more of the computation is stimulus driven, or bottom-up.

**Table 2:** Prediction of explicit judgment using free view, object search and saliency viewing fixation map respectively.

Blur level	1	2	3	4	5	6	7
Sal. View	.78	.86	**.87**	.86	.85	.85	.84
Free View	.76	.84	.**84**	.84	.84	.82	.81
Obj. Search	.74	.81	**.81**	.81	.79	.79	.79

The viewing pattern of observers is dependent on both top-down and bottom up influences. Top down influence may be expected to be greater in the object search task with bottom up influences dominating in the free viewing and saliency viewing tasks. In many cases, people view complex scenes having multiple objects or salient locations. When observers view a complex scene, they will fixate different locations based on their prior experience and random variability. For this reason, IOC scores for complex images are lower and the approximation performance of algorithms in predicting fixations is diminished.

## 3 Center bias

In the section, we discuss in detail the repercussions of center bias in saliency maps produced by popular algorithms, and a method to treat this bias. This form of bias interacts with the more commonly discussed image level, or fixation position bias that traditional ROC based saliency benchmarks have sought to control for.

### 3.1 Extent of center bias in saliency maps

The degree of center bias within data has been recognized as an important factor in performance evaluation. In a recent study, Borji *et al*. [4] proposed a center-bias ratio method to quantify the degree of bias in fixation data. This method assumes circles of different radii centered in the image and calculates the ratio of the number of fixation points inside the circle to the total number of fixations, for all possible circle sizes, providing a vector of ratio values that reflects the degree of central bias. Independent of the data, different saliency algorithms may exhibit different degrees of spatial bias due to their inherent computational structure. It is therefore of value to examine spatial bias among saliency algorithms in a manner analogous to the case for fixation data.

**Fig 3:**
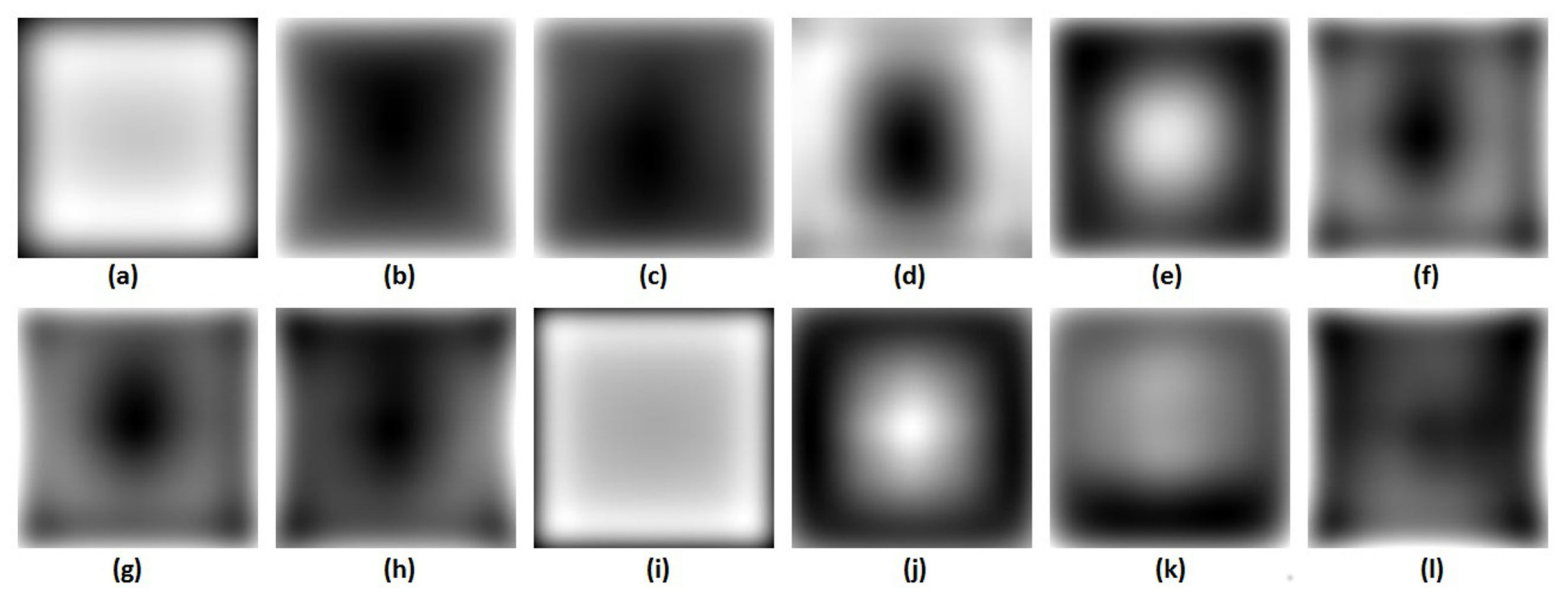
Spatial bias map of algorithms on Koehler *et al*. [2] dataset (a) Torralba [5], (b) HouCVPR [6], (c) HouNIPS [7], (d) AWS [13], (e) Itti-CIO2 [8], (f) ImageSignatureLab [9], (g) ImageSignatureRGB [9], (h) SDSR [10], (i) AIM [11], (j) GBVS [12], (k) Yan [14] and (l) SOC [15].

We visualize this bias as follows: Individual saliency maps for 12 different algorithms are first whitened to place the output values of different saliency maps on a common scale (and such that every saliency map has mean of 0 and standard deviation of 1). Suppose, an algorithm is applied to *N* images from a dataset produce saliency maps *M*_*i*_ where *i* = 1, 2, …, *N*. To whiten a saliency map, we calculate $M_{i}^{\prime }=\frac{M_{i}-\mu_{i}}{\sigma_{i}}$ where *μ*_*i*_ and *σ*_*i*_ are the mean and standard deviation of *M*_*i*_. The mean of all whitened saliency maps is given by $M^{\prime }=\frac{1}{N}\sum_{i=1}^{N}M_{i}^{\prime }$. Finally, the relative spatial bias of each algorithm is given by *M*_*bias*_ = *M*′ − *μ*_*m*_ where *μ*_*m*_ is the mean of *M*′. This *M*_*bias*_ provides a topological profile of relative spatial bias. This offers a sense of relative center bias in output produced by each algorithm. The overall spatial bias maps are shown in Fig 3. The spatial bias map is a topological profile that highlights regions that an algorithm tends to emphasize more than average in the saliency output relative to other algorithms. In this figure, high values within the center region (e.g. GVBS) imply strong relative central bias inherent in algorithm output and conversely, some algorithms (e.g. AWS) exhibit a relative peripheral bias. This provides a useful foundation for analysing differences in model performance across different types of data and also has implications for both standard and shuffled ROC scores. In particular, a strong relative center bias carries a predictable benefit for the standard ROC metric, however a stronger relative peripheral bias carries a benefit for the shuffled AUC metric.

### 3.2 Removal of center bias

If a saliency algorithm carries a strong center bias, then significantly higher values within the center region will appear consistently across all output maps. As stated before, because of the presence of center bias, the evaluation of saliency algorithms may be misleading. The term center bias removal technique refers to the strategies we have applied to remove the effect of center bias within different saliency maps. These techniques make the statistics of saliency values uniform at every pixel location across all saliency maps. In this paper, we consider two options to remove center bias in algorithm output to diminish sensitivity to spatial bias. In the current work, we propose two different processes of center bias removal that derives from the computational structure of the saliency models themselves.

#### 3.2.1 Rank order based center bias removal

**Fig 4:**
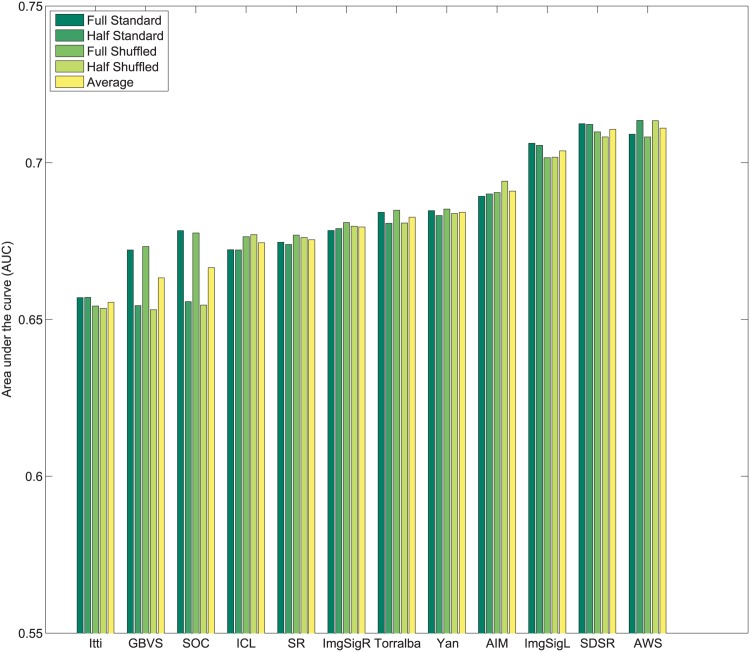
Rank order based center bias removal on Toronto dataset.

**Fig 5:**
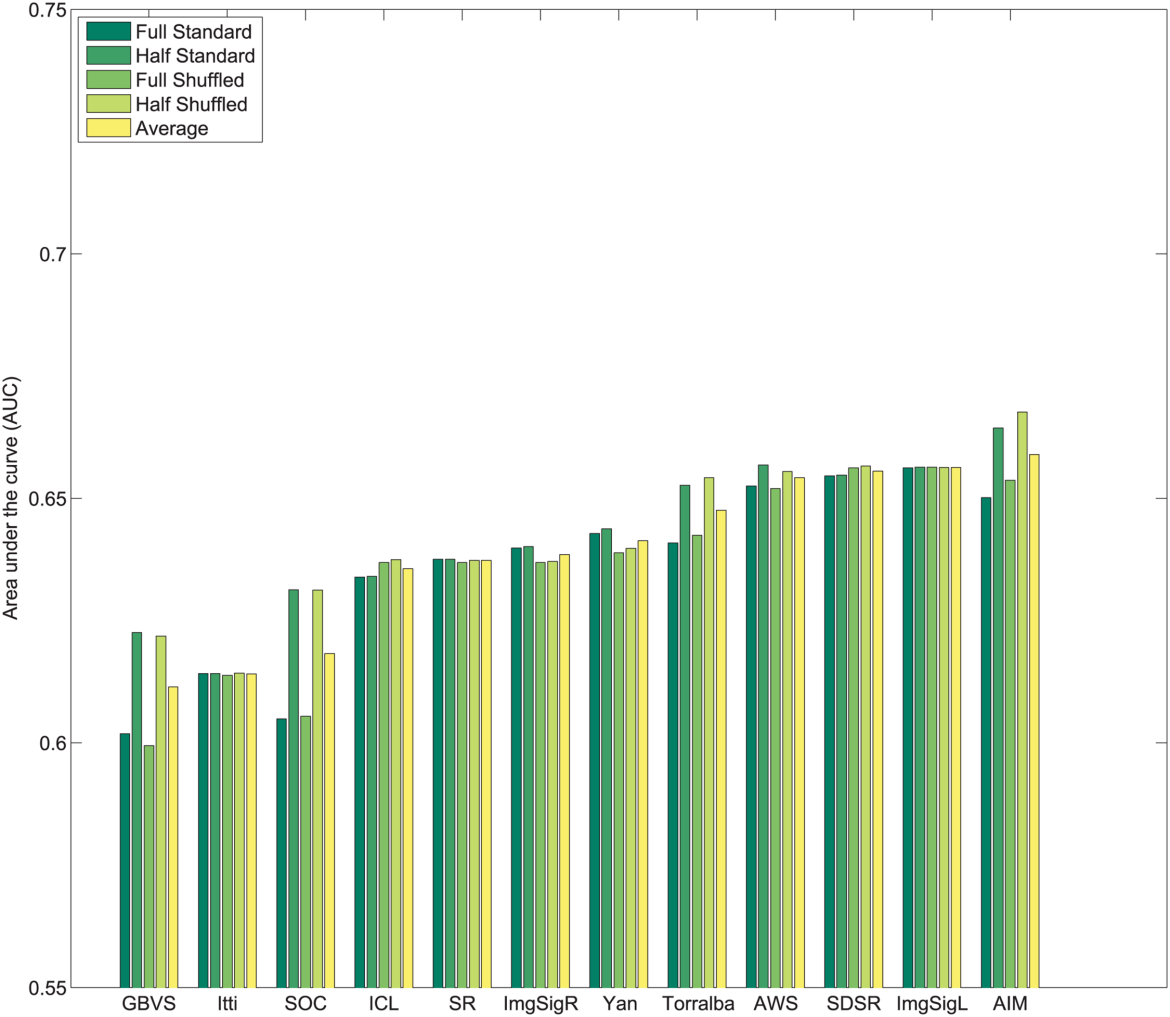
Rank order based center bias removal on Judd dataset.

**Fig 6:**
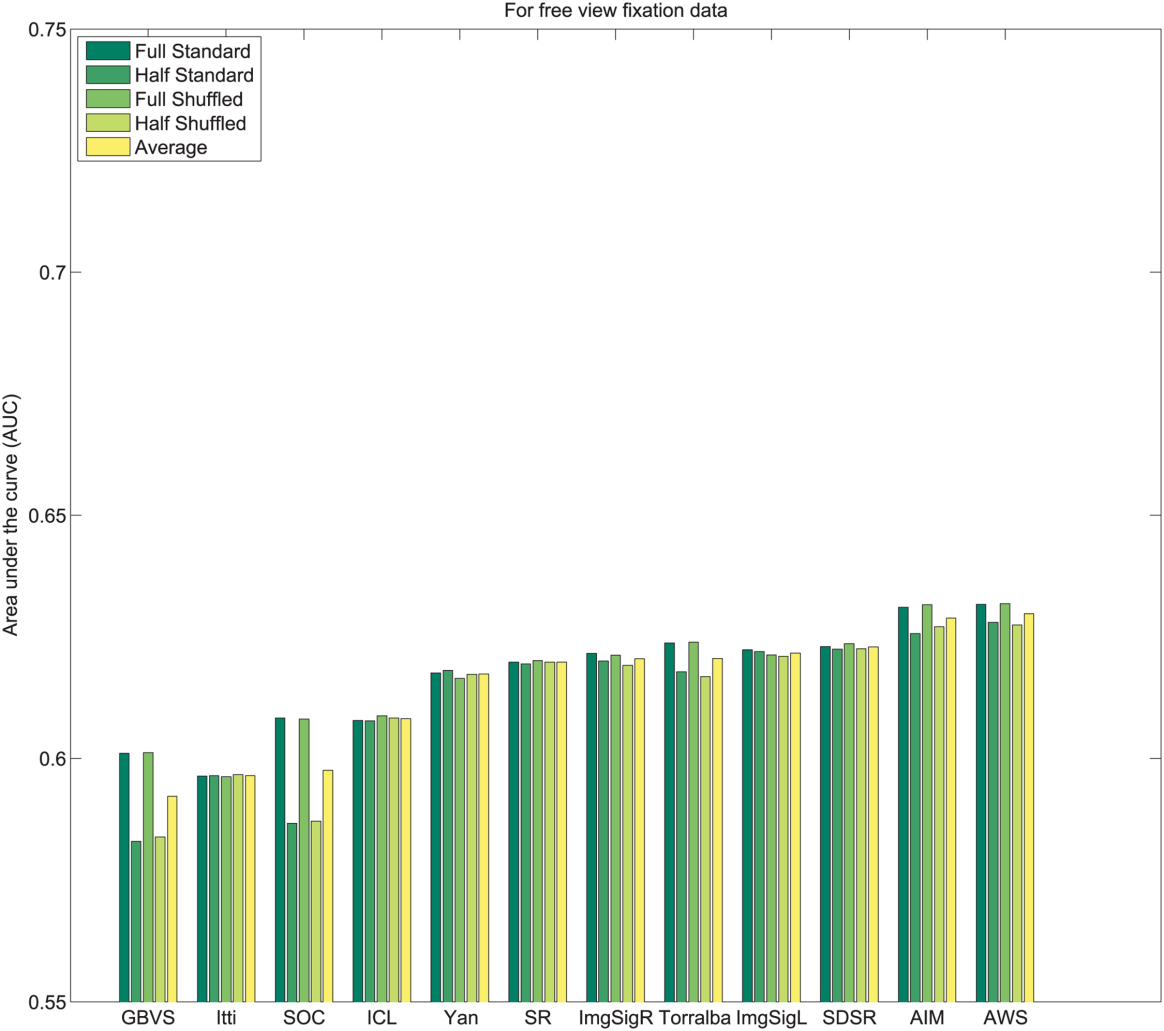
Rank order based center bias removal on Koehler *et al*. [2] dataset.

**Fig 7:**
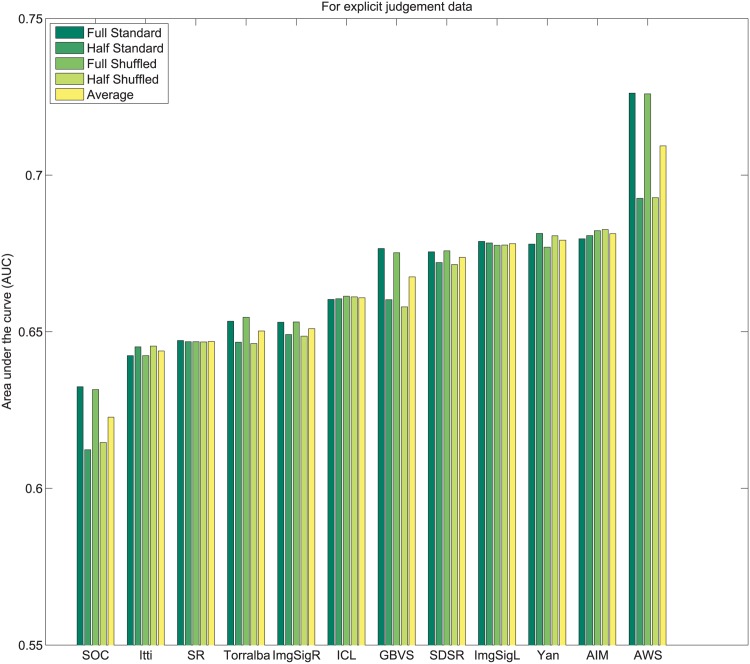
Rank order based center bias removal on Koehler *et al*. [2] dataset (Explicit Judgment).

To produce a uniform spatial distribution for the output of saliency algorithms, we wish to normalize the saliency values across all output maps on a per-pixel basis. We achieve this through the rank ordering of values in ascending order for a given pixel location across all saliency maps for the entire dataset. Suppose *M*_*i*_(*x*, *y*) is the pixel value corresponding to coordinate (*x*, *y*) in *i*th saliency map produced by any algorithm where *i* = 1, 2, 3, …*N*, and *N* is the total number of images considered for each algorithm. Now, for each location (*x*, *y*) we calculate the rank order of the *N* saliency values in *M*_*i*_. Subsequently, values of $\frac{1}{N},\frac{2}{N},\frac{3}{N},....1$ are assigned sequentially to the saliency maps based on this rank order. This imposes a uniform distribution of scores with the range $\frac{1}{N}$ to 1 across all maps, and for each pixel location. Following this center bias removal from all images of the dataset, the sum of all values corresponding to a given pixel location across all saliency maps is $\sum_{i=1}^{N}M_{i}(x,y)=\frac{N+1}{2}$. After spatial center bias removal, saliency output may be evaluated using both standard ROC [3] and shuffled ROC [4, 16] analysis. The rankings of algorithms after removal of spatial bias in output on Toronto [11], Judd *et al*. [3] and Koehler *et al*. [2] datasets are shown in Figs 4–7 respectively.

#### 3.2.2 Whitening based center bias removal

**Fig 8:**
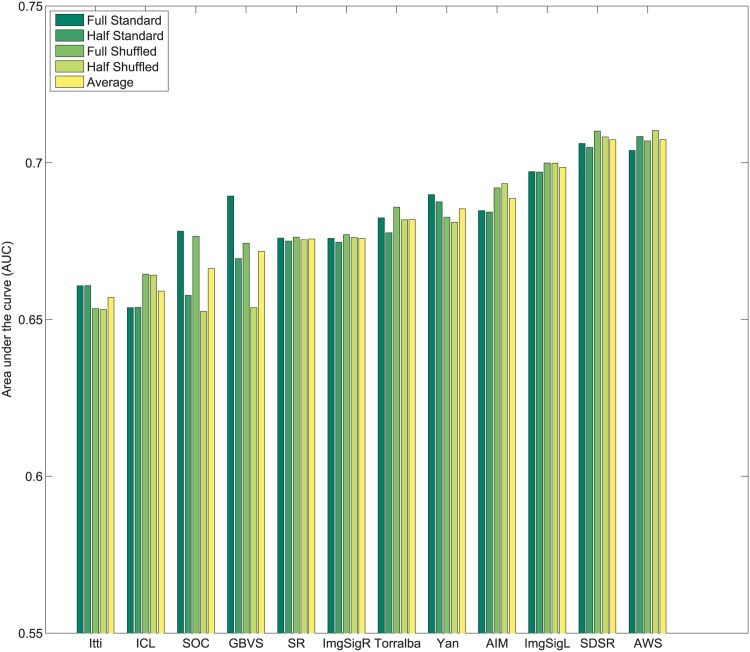
Whitening based center bias removal on Toronto dataset.

**Fig 9:**
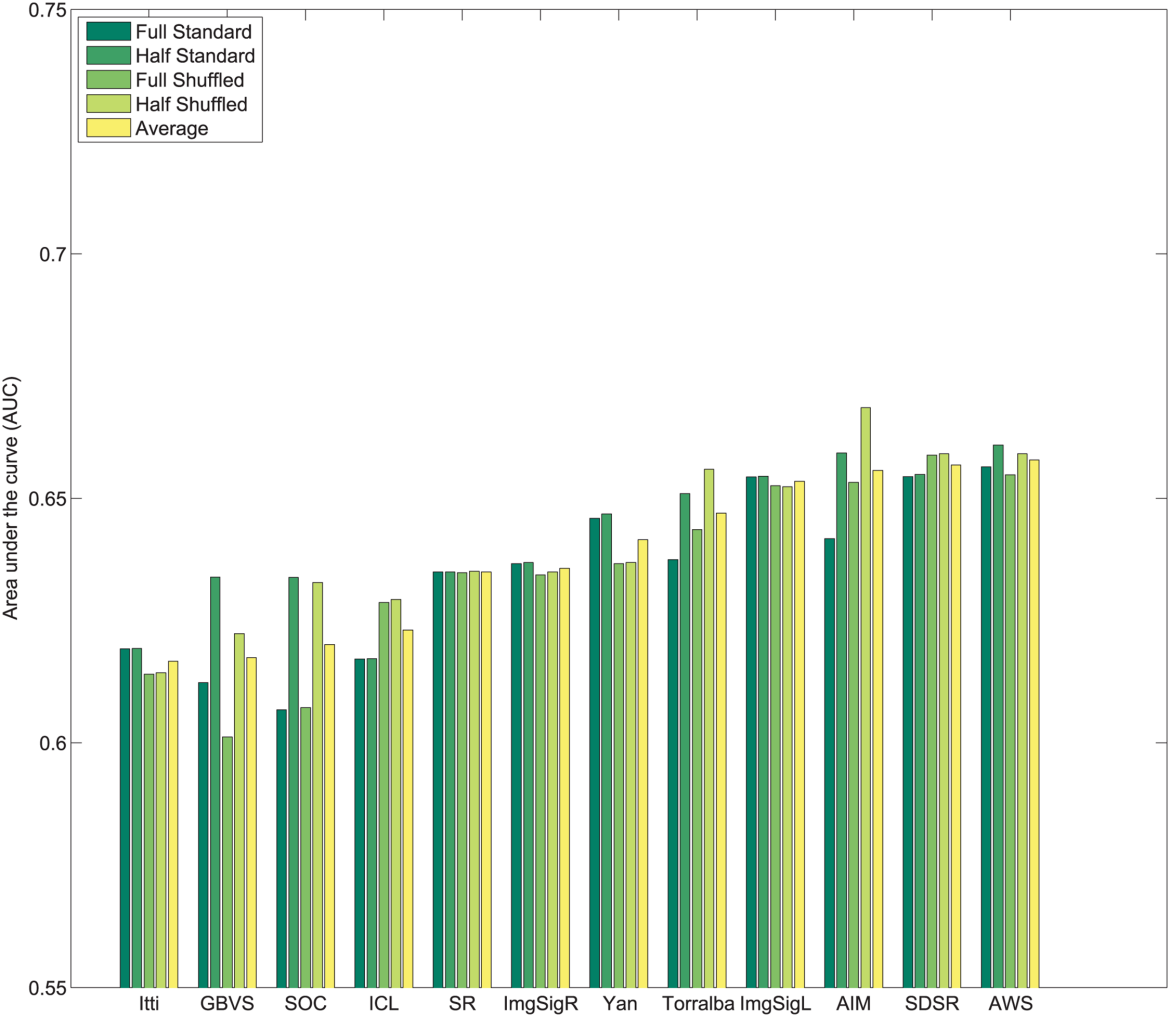
Whitening based center bias removal on Judd dataset.

**Fig 10:**
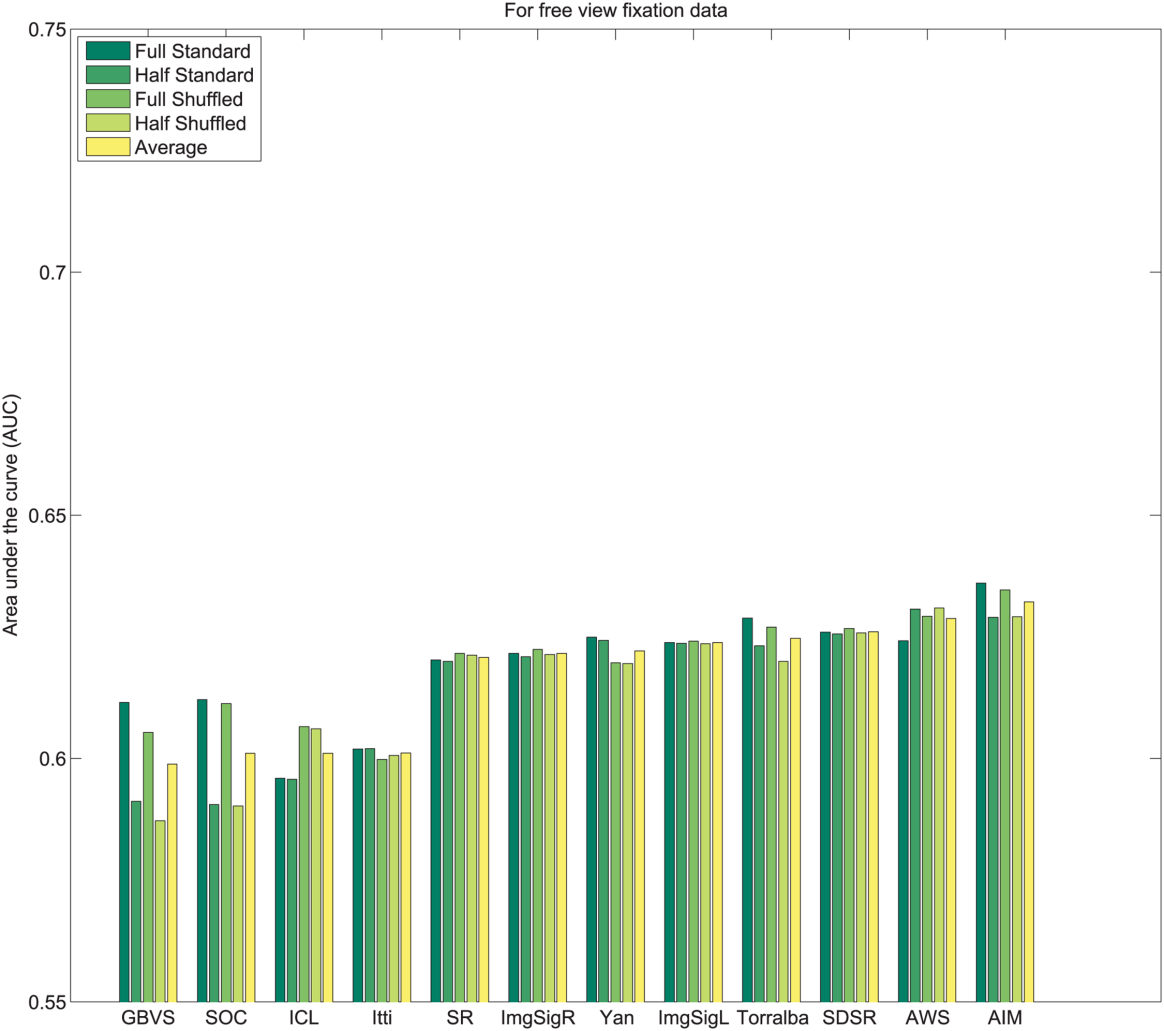
Whitening based center bias removal on Koehler *et al*. [2] dataset.

**Fig 11:**
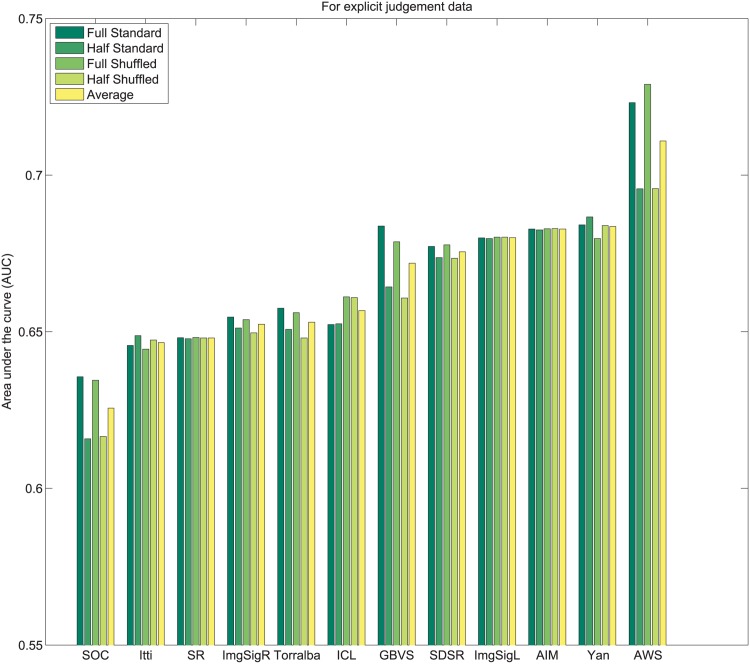
Whitening based center bias removal on Koehler *et al*. [2] dataset (Explicit Judgment).

An alternative to rank ordering, is to apply a more standard statistical whitening operation. First, individual saliency maps are whitened to produce maps with zero mean and unitary standard deviation. Subsequently, pixel-wise whitening is carried out across the saliency maps for each individual pixel location. After this operation $\sum_{i=1}^{N}M_{i}(x,y)=0$ and each algorithm carries a 0 mean and unit variance across all saliency maps for each pixel location. The ranking of algorithms after center bias removal using this process on Toronto [11], Judd *et al*. [3] and Koehler *et al*. [2] datasets are shown in Figs 8–11 respectively.

A few observations related to both strategies for center bias removal are as follows:

- Resizing the input images changes the scale space spanned by the features for some algorithms, which may alter performance. Sensitivity to scale is a factor that is important to control for outside of spatial bias.
- Both standard AUC analysis [3] and shuffled AUC analysis [16] produce very similar results for a fixed image scale with normalized outputs.
- In contrast to the standard ROC benchmarks, spatial bias removal for algorithm output produces more consistent performance of algorithms across different datasets, and metrics.

## 4 Explicit judgment prediction

In this section, we focus on prediction of explicit judgment. There are two goals that are central to this section. The first goal is to determine how well existing models of visual saliency are able to predict locations selected through explicit judgment. While some sense of this is already provided in our benchmarking results, we further examine the strength of predictions that may be achieved through an ensemble approach that relies on existing saliency algorithms. This provides a new standard for future efforts in prediction of explicit judgment data, but also informs on how significant the difference is between models tuned to perform well on fixation data, and performance that is possible for explicit judgments. A second important goal of this section is to relate explicit judgment data to fixation data through a predictive model. Fixation data is ubiquitous, while measurement of explicit judgments of visual saliency is relatively rare. These two types of experimental data offer different vantage points on the saliency of image content to a human observer. There is value to each type of data in differences in qualitative analysis that is possible through heatmaps or other visualizations, and quantitative analysis in benchmarking and predictive models. We therefore examine the extent to which existing eye tracking data can be used to simulate the distribution that one would expect from an explicit judgment experiment. Given that both types of data are available within the Koehler *et al*. [2] dataset, this also allows for the relationship between the two types of data to be examined.

### 4.1 Prediction model

A goal of prediction in this context, is to establish the extent to which explicit judgment prediction may be improved beyond existing standard saliency models while relying on similar principles and features. From the benchmarking results in Table 1, we know that individual saliency algorithms can approximate explicit judgment to a certain degree. If we constrain our own prediction model to the space of features, and saliency measures spanned by existing models of visual saliency, this provides an indication of how well principles driving existing saliency models translate to prediction of explicit judgment data. This evaluation also succeeds in setting an improved benchmark score for explicit judgment prediction. To achieve these goals, an ensemble prediction based on a range of saliency models is used.

Before delving directly into performance of our ensemble classifier for explicit judgment prediction, it is useful to further consider task relatedness for fixation tasks, and explicit judgments. If data from any of the fixation conditions is converted to a continuous density map via Gaussian blurring (as is standard in saliency evaluation), performance in predicting explicit judgments varies as a function of the degree of blur. Comparing saliency output subject to different degrees of blurring is a standard practice for saliency evaluation [4, 9]. Prior research efforts demonstrate that performance of any saliency algorithm is highly sensitive to the degree of post-processing Gaussian blur. To ensure that comparison among different types of saliency maps is subject to a fair comparison the degree of blur that corresponds to best performance is considered. If we do not consider such blurring or only consider a low level of blurring the distinction in performance across algorithms is less clear. Approximation of explicit judgment locations by fixation data from the three different fixation tasks is shown in Table 2. Both free viewing and saliency viewing fixation maps can approximate explicit judgment quite well, and better than object search fixations. In the object search task, although observers are directed to find a pre-defined object, the wide variety of objects presented in a typical scene and influence of visual saliency nevertheless results in fixations on salient items in the scene even though they may not be task relevant. The relationship to explicit judgment indicates that there is indeed a significant degree of task-independent commonality between free viewing, saliency viewing and explicit judgment.

We have also examined how the results appearing in Table 2 interact with IOC on a per-image basis. To accomplish this we compare the base IOC scores (per image) within each category of fixation data with the standard AUC performance in predicting explicit judgments from the fixation data. This provides an understanding of the relationship between IOC within each type of fixation data, and the relatedness of fixation and explicit judgment data as a function of IOC. This is measured by examining the Pearson correlation on a per image basis between image IOC for the fixation data, and the standard AUC for the prediction of explicit judgments from fixations. Correlation values from this line of experimentation are as follows:

- Free viewing IOC vs. standard AUC for prediction of explicit judgment from free viewing data: 0.41
- Object search IOC vs. standard AUC for prediction of explicit judgment from object search data: 0.29
- Saliency viewing IOC vs. standard AUC for prediction of explicit judgment from saliency viewing data: 0.51

The ability to predict explicit judgment based on fixations is good when the IOC for the fixation data associated with the same image is high. Statistically, this result indicates that when observers exhibit more similarity in their fixations for a particular image, then factors that drive the selection of fixated locations and explicit judgments also become less disparate. This hints at overlap in factors driving the two processes, and also that per-image confidence for simulating explicit judgment data from fixations, might be measured through the associated IOC score.

As mentioned, a second goal in this section is the simulation of explicit judgment data when only eye tracking data is available. From Table 2, it is evident that fixation data is also indicative of locations selected in the explicit judgment task. Thus, it is natural to consider the combined strength of fixation data and saliency algorithms in simulating a measure of explicit judgment. In line with this goal, we consider only free viewing fixation data given that this tends to be the standard data type that is widely available.

**Fig 12:**
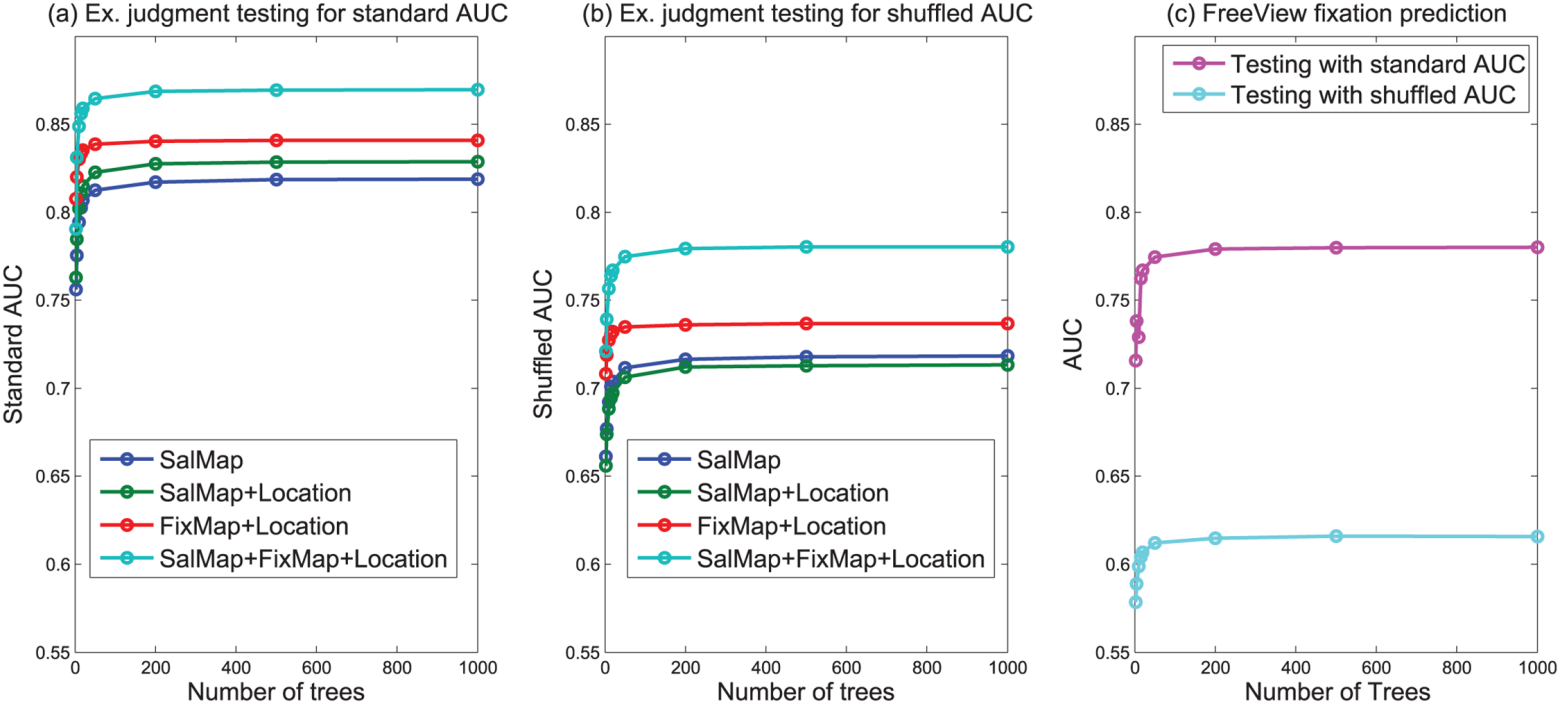
Performance for explicit judgment prediction. (Best viewed in digital format).

**Fig 13:**
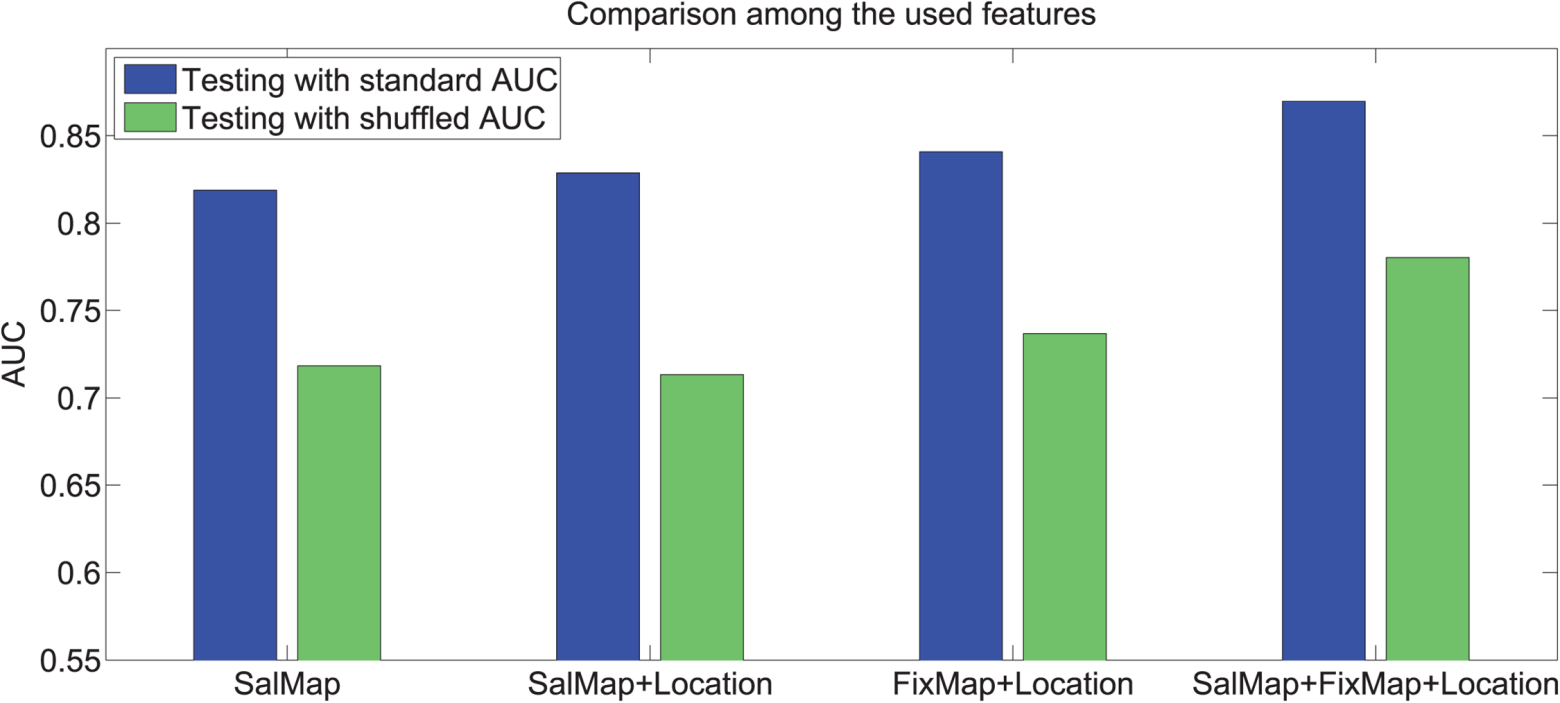
Explicit judgment prediction using different feature sets. (Best viewed in digital format).

To train the prediction model, the image set with explicit judgment ground truth is divided into training and test sets (half/half). For each image, there are 100 locations selected as most salient through explicit judgment and these are labelled as positive samples. From each image, we also randomly sample locations not among the positive samples as negative samples. Output values from 12 saliency maps for the positive and negative pixel locations are sampled as training data. In addition, in support of the simulation of explicit judgment, fixation density from blurred fixation maps are sampled as an additional statistic. Given that there may also be dependency on spatial location, and also on the spatial dispersion of positive samples across task, we also include the (x,y) coordinates of samples as additional statistics. Features from all the images, are used to train an ensemble of bagged decision trees for regression (Random Forest [19]). For testing, saliency values and fixation densities for each pixel location are then used to predict explicit judgments. Standard and shuffled AUC scores, subject to a varying number of trees are shown in Fig 12 (a) and 12(b). Fig 12 includes the decomposition into different combinations of feature types, including only saliency output, only fixation data, and combinations thereof. Note that center bias removal is not applied here as most of the cases explicitly include spatial position as a feature. This illustrates the extent to which existing saliency algorithms are capable of predicting locations selected in explicit judgment, and also addresses the extent to which explicit judgments may be simulated from fixation data with saliency algorithms as an adjunct source of information. Fig 13, demonstrates differing degrees of performance possible under different conditions or through different combinations of feature types.

As a baseline for assessing the boosted classification performance of saliency algorithms in predicting explicit judgment locations, it is useful to also examine how much a boosting approach improves performance for the traditional fixation based evaluation. Experiments measuring this case follow the same process described for explicit judgment prediction, in using ensemble performance across saliency algorithms to predict fixated locations. Results relating to these experiments appear in Fig 12(c). We find that although a boosted classifier based on saliency algorithms is able to significantly improve the performance of explicit judgment prediction, there are no significant gains in fixation data prediction (refer to Table 1 to make this comparison). This suggests that similar factors are involved in determining explicit judgment locations and fixations, however models have been more finely tuned to characteristics of fixation data (e.g. spatial dispersion / distribution).

### 4.2 Explicit judgment performance prediction

**Fig 14:**
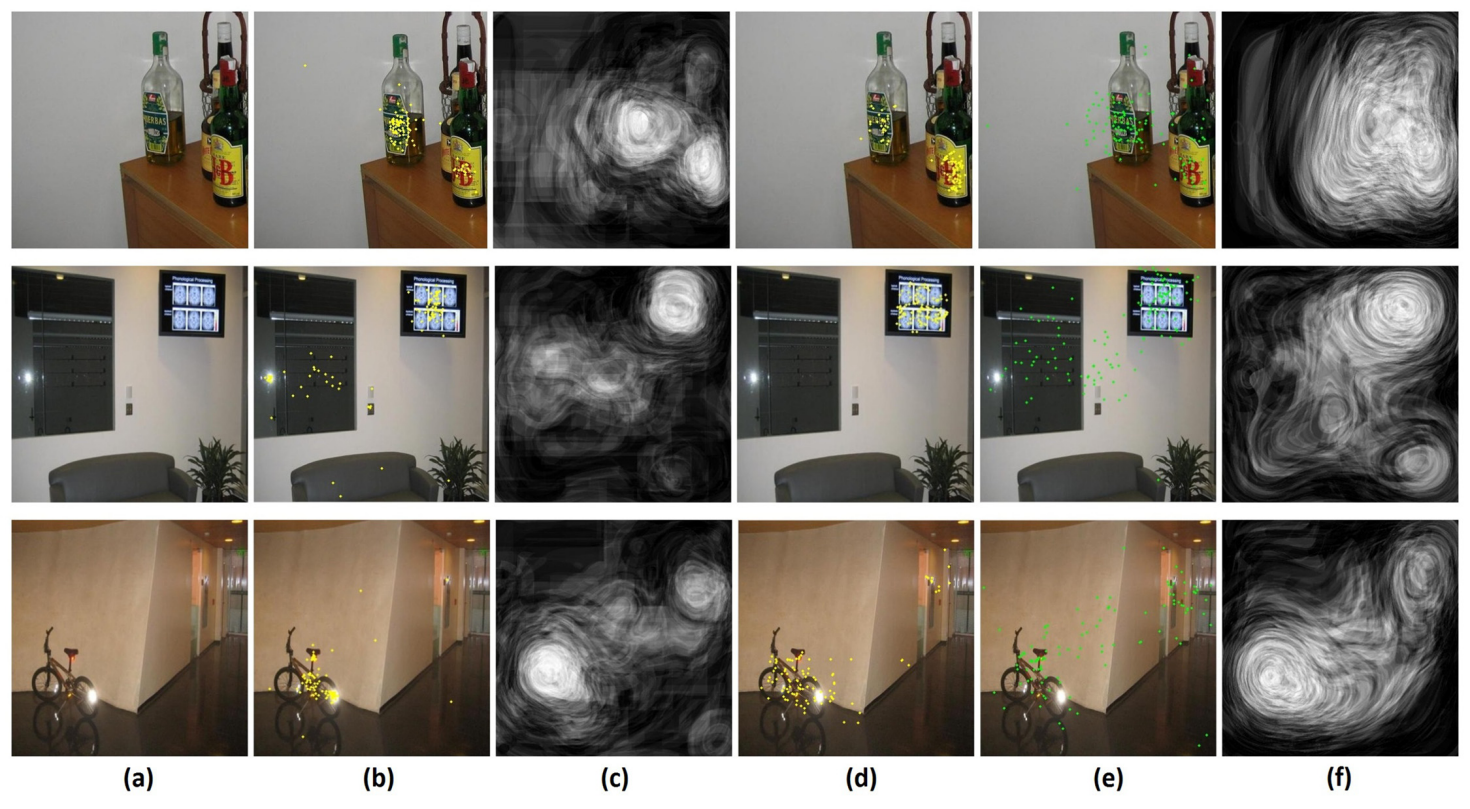
Sample output column (a) input image (b) ex. judgment locations (c) predicted ex. judgment map (d) simulated ex. judgment locations (e) free view fixation locations (f) predicted fixation map.

A sample of input images and their associated actual explicit judgments, predicted explicit judgments (as a saliency map) and simulated explicit judgment data are presented in Fig 14. This also includes the true fixation data and predicted fixations (traditional saliency) for comparison.

Fixation data from a free viewing task is the most common form of data in evaluating visual saliency. However, there are evidently benefits to having the ability to leverage explicit judgment data in guiding algorithm development and assessing performance. In this section, we assess whether the prediction of explicit judgment made possible by our model is of sufficient quality that this output may be used in a surrogate role for quantitative assessment. For example, given a new algorithm for visual saliency prediction, and various fixation data sets, can one predict the relative performance of the new algorithm for the explicit judgment task when only fixation data is available.

The predicted explicit judgment map consists of a topological representation of expected explicit judgment locations. Evaluation, whether by fixations or explicit judgment typically relies on binary values corresponding to discrete pixel locations. To generate this type of representation for quantitative evaluation on simulated explicit judgment data, we first normalize the predicted explicit judgment map (saliency output). Subsequently, 100 randomly sample locations are chosen based on the values in the predicted explicit judgment map, ignoring locations where the value within the explicit judgment map falls below a set threshold. This strategy was found to produce superior results to non-thresholded sampling from the predicted explicit judgment map. Given discrete coordinates for simulated explicit judgment data, evaluation may proceed based on the standard methods for ROC analysis.

Saliency algorithms were evaluated based on free viewing fixation data, explicit judgment data, and simulated explicit judgment data. The critical factor in this analysis is the relation of performance on true explicit judgment data to simulated explicit judgment data. The correlation between ROC scores of different algorithms for true explicit judgment data, and simulated explicit judgment data is 0.92. In contrast, correlation between per image ROC scores in predicting fixation data and in predicting explicit judgments is 0.46. This provides confidence that the simulated explicit judgment data derived from fixation data may present a suitable approximation for analyzing relative algorithm performance for explicit judgment tasks when only eye tracking data is available, and that saliency is necessary to provide a bridge between these disparate sources of data.

### 4.3 Details of post-processing blur for ensemble prediction

As with most benchmarking efforts in visual saliency prediction, performance is affected by the amount post-processing blur. This effect is presented in Table 3.

**Table 3:** For a fixed NTree = 15, the effect of different Gaussian blur level in the explicit judgment prediction model.

Blur level	1	2	3	4	5	6	7
Standard AUC	.83	.85	.85	.86	.85	.85	0.84
Shuffled AUC	.73	.74	.76	.76	.76	.75	.73

### 4.4 Saliency and Segmentation

Given that explicit judgment locations are likely to be under greater cognitive control than early fixations, explicit judgment location may arguably provide a stronger marker for object locations than fixation data. To evaluate this hypothesis, we have carried out experiments involving object segmentation performance based on both predicted explicit judgment locations and predicted fixations using 200 images including one and two object cases from the SED dataset [20]. To evaluate segmentation performance, we have calculated the F-measure by the following equation: $\text{F-measure}=\frac{precision\times recall}{0.5\times (precision+recall)}$

**Fig 15:**
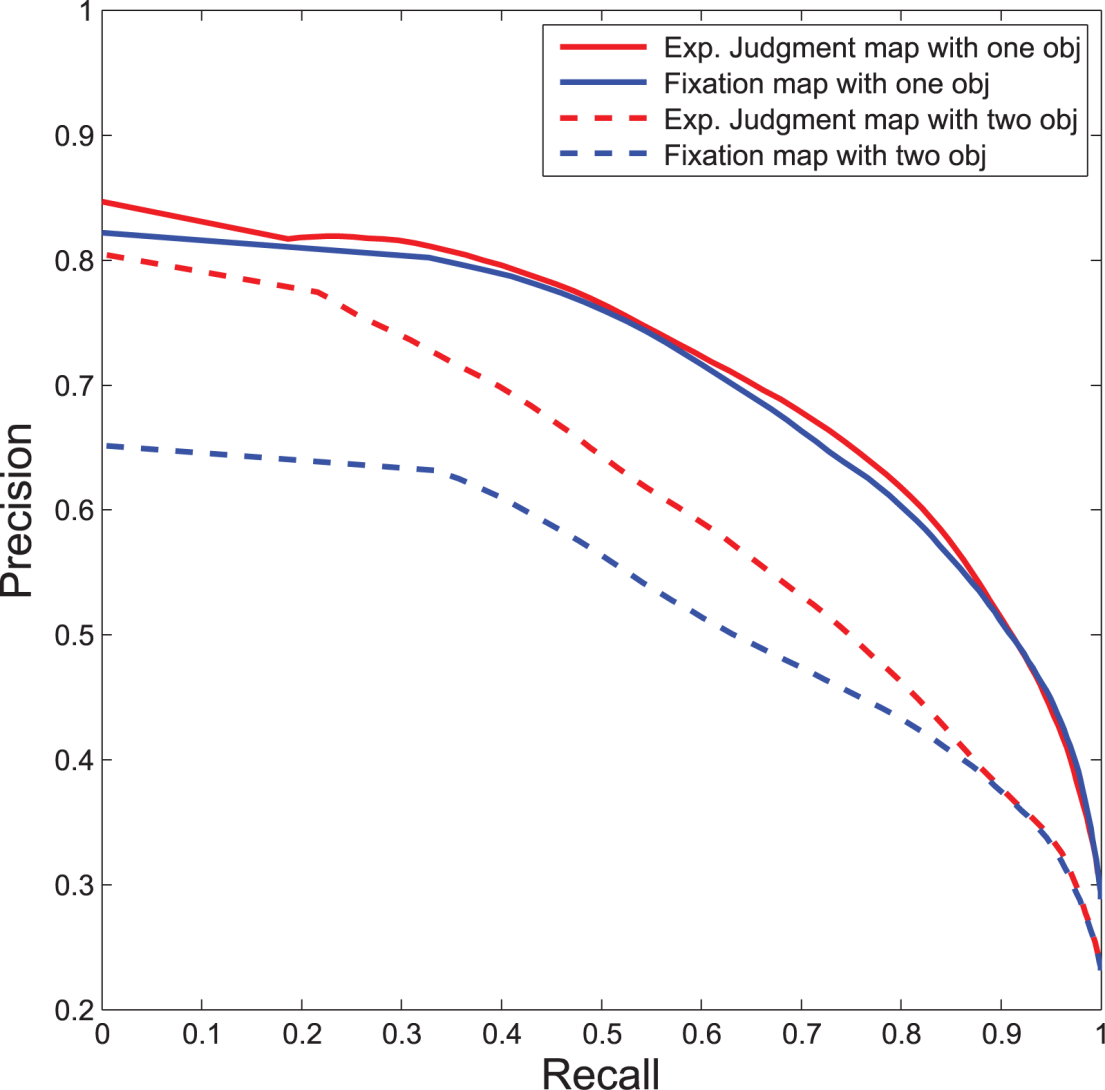
Precision-Recall curve for one and two object cases using for ensemble classifiers trained on explicit judgment (red) and fixation data (blue) respectively.

F-measure scores for the overall dataset are shown in Table 4 and the corresponding precision-recall curves in Fig 15. These results indicate that predicted explicit judgment maps are more successful than predicted fixation maps in highlighting segmented object locations within a scene. Moreover, the performance improvement using predicted explicit judgment maps over predicted fixation maps in the two object case is higher than that of one object. This also suggests that the distinction between predicted explicit judgment maps vs. fixation maps becomes more prominent for complex scenes having multiple objects.

**Table 4:** F-measure of segmentation performance.

Object case	Exp. judgment map	Fixation map
One object	0.698	0.689
Two object	0.604	0.566

## 5 Conclusion

In this paper, we address the problem of computational modeling of visual saliency in considering explicit judgment data, as opposed to the more common alternative of predicting fixations within human gaze data. There are a number of contributions and results of importance that are derived from this investigation:

1. Benchmarking results are presented for several algorithms across four different tasks. This demonstrates the performance landscape of popular algorithms for visual saliency for explicit judgment of salient locations. We also motivate why this alternative form of ground truth may be advantageous for perception related predictions and computer vision applications.
2. The experimentation included also provides an indication of task relatedness. Free viewing and especially saliency viewing appear to be driven by factors that overlap with explicit judgment. With that said, there are different confounding factors associated with each type of data, and reason to believe that explicit judgment is more proximal to representing the quality that saliency algorithms aim to predict.
3. Results indicate that IOC predicts the similarity of fixation data to explicit judgment data. This implies that IOC may be a reliable measure for gauging the extent to which vision is stimulus driven, and how well explicit judgment may be inferred from fixations.
4. We present an alternative to existing benchmarking strategies to simultaneously normalize for bias in data and algorithm output. This produces a relatively consistent ranking of saliency algorithms across different data sets and tasks.
5. Existing algorithms designed for fixation prediction do reasonably well in predicting explicit judgments. However, these algorithms perform much better when tuned to predict this data specifically. This implies that underlying principles in existing saliency algorithms are suitable for predicting explicit judgment, but that some optimizations of algorithm performance have been specific to statistical properties of the spatial layout of fixation data.
6. Explicit judgment marks an alternative window into perceptually important content to human observers. While eye tracking data is relatively standard in many research domains, explicit judgment tasks are not. We have also presented a benchmarking result that surpasses current saliency algorithms for explicit judgment prediction, in addition to a method that allows for fixation data and saliency models to approximate explicit judgment data to an extent that qualitative interpretation and quantitative analysis towards explicit judgment prediction may be carried out when only fixation data is available.

As a whole, this work establishes performance standards for the explicit judgment task, introduces an alternative ROC based benchmark strategy, demonstrates the relatedness of perceptual tasks, and provides methods for data simulation across different perceptual tasks.
